# The combined utilization of predictors seems more suitable to diagnose and predict rotator cuff tears

**DOI:** 10.1186/s12891-022-05986-3

**Published:** 2022-11-25

**Authors:** Qi Ma, Changjiao Sun, Hong Gao, Xu Cai

**Affiliations:** 1 Department of Orthopaedics, Beijing Tsinghua Changgung Hospital, School of Clinical Medicine, Tsinghua University, No.168, Litang Road, Changping District, Beijing, 102218 China; 2 Beijing MEDERA Medical Group, Beijing, China

**Keywords:** Acromion, Diagnosis, Humeral greater tuberosity, Prediction, Rotator cuff tear, Three-dimensional imaging

## Abstract

**Background:**

Morphological markers presenting the lateral extension of acromion and the greater tuberosity of humerus were proposed to diagnose and predict rotator cuff tears (RCTs) in recent years, but few studies have addressed the combined performance when using two predictors together. As a presence of a RCT may be associated with the impingement caused by both acromion and the greater tuberosity, we believe a combined utilization of predictors could result in a better diagnostic and predictive performance than using a single predictor. The aim of this study is to (i) explore whether the combination is more efficient to predict and diagnose RCTs; (ii) find out which combination is the most superior screening approach for RCTs.

**Methods:**

This was a retrospective study and patients who visited our hospital and were diagnosed with or without partial-thickness or full-thickness RCTs via magnetic resonance imaging from January 2018 to April 2022 were enrolled and classified into two groups respectively. Four predictors, the critical shoulder angle (CSA), the acromion index (AI), the greater tuberosity angle (GTA) and the double-circle radius ratio (DRR) were picked to participate in the present study. Quantitative variables were compared by independent samples t tests and qualitative variables were compared by chi-square tests. Binary logistic regression analysis was used to construct discriminating combined models to further diagnose and predict RCTs. Receiver operating characteristic (ROC) curves were pictured to determine the overall diagnostic performance of the involved predictors and the combined models.

**Results:**

One hundred and thirty-nine shoulders with RCTs and 57 shoulders without RCTs were included. The mean values of CSA (35.36 ± 4.57 versus 31.41 ± 4.09°, *P* < 0.001), AI (0.69 ± 0.08 versus 0.63 ± 0.08, *P* < 0.001), DRR (1.43 ± 0.10 versus 1.31 ± 0.08, *P* < 0.001) and GTA (70.15 ± 7.38 versus 64.75 ± 7.91°, *P* < 0.001) were significantly higher in the RCT group than for controls. Via ROC curves, we found the combined model always showed a better diagnostic performance than either of its contributors. Via logistic regression analysis, we found the values of both predictors over their cutoff values resulted in an increasement (20.169—161.214 folds) in the risk of having a RCT, which is more than that by using a single predictor only (2.815 -11.191 folds).

**Conclusion:**

The combined utilization of predictors is a better approach to diagnose and predict RCTs than using a single predictor, and CSA together with DRR present the strongest detectability for a presence of RCTs.

## Background

Rotator cuff tear (RCT) is the most common disorder of shoulder nowadays and is characterized by shoulder pain and limitation of shoulder activity. A complex multifactorial process involving intrinsic and extrinsic factors contributes to a presence of a RCT. However, there still remains controversy on which factor is primary or secondary. From an intrinsic aspect of view, tensile overload, aging, microvascular supply and traumatism result in a degenerative and fragile tendon, making the tendon tear more easily [1]. From an extrinsic aspect of view, anatomic variables such as the hooked acromion and morphology of coracoacromial ligament narrow the subacromial space and increase pressure on tendons when lifting the upper limbs, leading to teared tendons due to the impingement between bony structures [1, 2].

For a patient complaining of shoulder discomfort but with a negative magnetic resonance imaging (MRI), early detection and prediction of a presence of RCTs are important to help the patient avoid suffering from a potential progression of cuff injury in the future and thus reduce the spending related with hospitalization and operation. Based on the mechanisms of extrinsic factors, predictors measured in a true anteroposterior view of shoulder were proposed to predict and diagnose RCTs in previous researches, most of which were centered around acromion, such as the acromion index (AI) and the critical shoulder angle (CSA), with a higher value corresponding to a more laterally extended acromion and correlating with a higher risk of developing RCTs [3, 4]. In recent studies, researchers started to realize the greater tuberosity of humerus also might play a vital role in the occurrence of RCTs as the impingement was formed by both acromion and the greater tuberosity, suggesting the greater tuberosity should be equally important as acromion. Two representative predictors, the greater tuberosity angle (GTA) and the double-circle radius ratio (DRR), were capable to measure the superolateral extension of the greater tuberosity and found to be practical and reliable to detect RCTs in current researches [5, 6]. It is a great advancement that clinicians focus not only on acromion but also on the greater tuberosity to try to understand the mechanisms and reveal the whole pathological process of RCTs.

However, the detectability of those predictors for RCTs was usually discussed individually, either from a single perspective of the acromion, or from a single perspective of the greater tuberosity. As both the acromion and the greater tuberosity contribute to the development of RCTs, we infer that a combination of a predictor evaluating the acromial morphology and another assessing the morphology of the greater tuberosity could be more superior to predict and diagnose RCTs than either alone. To our best acknowledge, few researches discussed the combined diagnosis and prediction of RCTs and we could only find one paper on this topic, where conclusion was drawn that a summation of CSA and GTA values over 103° increased the odd of having a RCT by 97 folds and was efficient to diagnose RCTs with a sensitivity of 91% and a specificity of 92% [7]. To provide a supplement in this field, we picked out four predictors, two from the acromion (CSA and AI) and another two from the greater tuberosity (GTA and DRR), to participate in the present research and aimed to (i) explore whether the combination is more efficient to predict and diagnose RCTs than using a single predictor; (ii) find out which combination is the most superior screening approach for RCTs. We hypothesize that the combined utilization of two predictors could show a better performance of predicting and diagnosing RCTs, and the combination of CSA and DRR is the best solution among all combinations because CSA is a direct reflection of acromial extension, and the measurement of DRR is more reliable because it does not refer to the morphology of humeral diaphysis.

## Methods

### Patients

This was retrospective research. Partial-thickness and full-thickness RCTs were all included in our study. A partial-thickness tear was defined by the articular-sided or bursa-sided hyperintensity observed within rotator cuff on T2-weighted fat suppression MRI. A full-thickness tear was defined by complete rupture or retraction of the tendon observed on T2-weighted fat suppression MRI. The inclusion criteria were (i) patients who visited the orthopedic department at our hospital because of symptomatic shoulder disorders, and those who were admitted to our trauma center because of blunt trauma including strike, fall, pulling and sudden stretch around shoulders from January 2018 to April 2022; (ii) definitely diagnosed with or without partial-thickness or full-thickness RCTs via MRI; (iii) not combined with other shoulder disorders such as tendinosis, osteoarthritis or neoplasm; (iv) computed tomography (CT) of affected shoulder joint was performed with arm in neutral rotation. The exclusion criteria were (i) previous history of fractures, dislocations or operations around shoulders; (ii) incomplete demographic information; (iii) patients with negative MRI but complaining symptomatic shoulder disorders as a result of trauma; (iv) scapular glenoid versions larger than ± 10°; (v) patients younger than 40 years old. The cohort consisting of patients with RCTs were classified into the RCT group, and those without RCTs were classified into the control group.

### Ethics and consent

Our study was approved by the institutional review board of Beijing Tsinghua Changgung Hospital (No. 22303–6-01). All procedures involving human participants were in accordance with the declaration of Helsinki and informed consents were obtained from individual participants included in the study.

### Measurements

We used the United Imaging Medical Processing Software (uWS-CT, United Imaging, Shanghai, China) to analyze the CT images with slice thickness of 1.0 × 0.8 mm. Through multiplanar reconstruction we got a complete shoulder joint in three-dimensional (3D) vision. All measurements in this study were carried out on these 3D models.

#### (i) Establishing a coordinate system

**Fig. 1:**
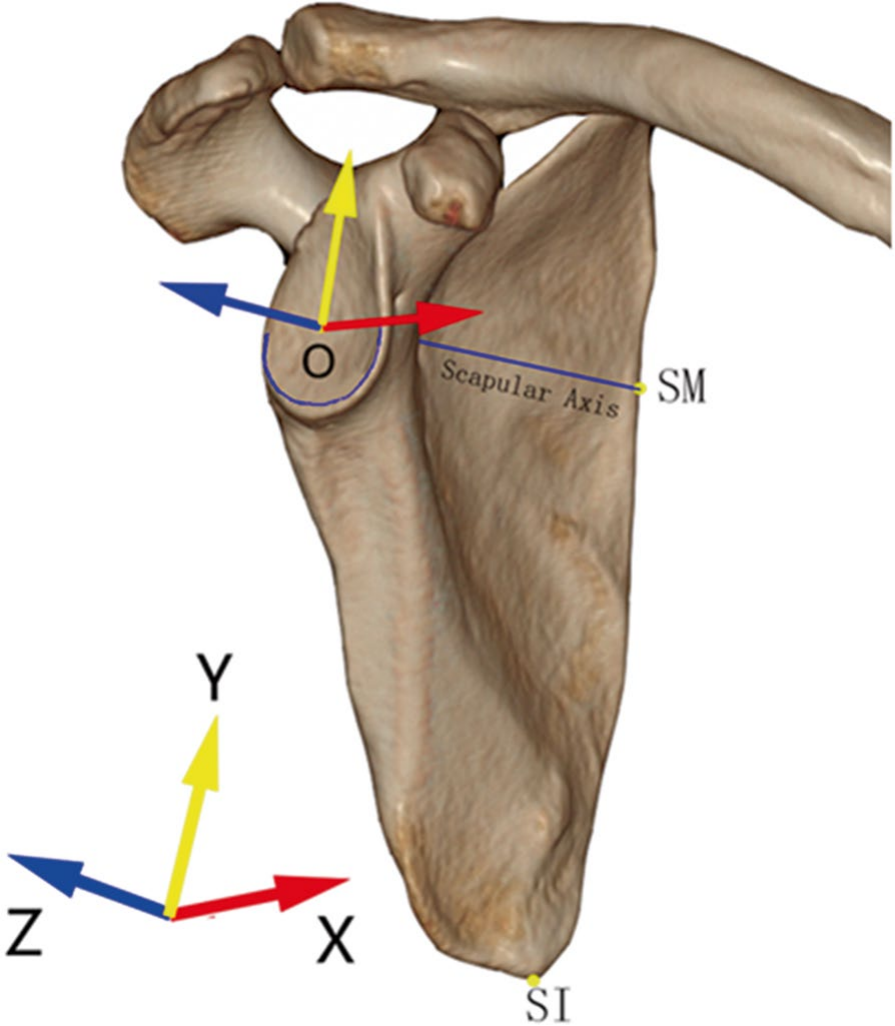
The coordinate system based on scapula. The center of the best-fit circle of the inferior glenoid (O) was as the origin. The line from O to SM was set as Z-axis (blue). The plane determined by O, SM and SI was regarded as YZ plane. X-axis (red) was perpendicular to the YZ plane and Y-axis (yellow) was perpendicular to the XZ plane

A coordinate system was established via the rationale described in Karns et al.’s and Suter et al.’s literatures, [8, 9] and the procedures had been described in previous research as follows: We defined the center of the best-fit circle of the inferior glenoid as the origin (the point O). The line connecting the origin and the point where the scapular spine intersected the medial border of the scapula (SM) was set as Z-axis. The plane determined by the Z-axis and the most inferior point on the inferior scapular angle (SI) was defined as YZ plane. The line starting from the origin and perpendicular to the YZ plane was X-axis, and the line beginning from the origin and perpendicular to the XZ plane was Y-axis (Fig. 1). By rotating scapula around the Y-axis to correct the glenoid version, we could get a viewing perspective with an overlap of the anterior and posterior contour of the glenoid when looking perpendicular to the YZ plane, which was thought to resemble the true anteroposterior view of shoulder joint [6].

#### (ii) Double-circle radius ratio (DRR)

**Fig. 2:**
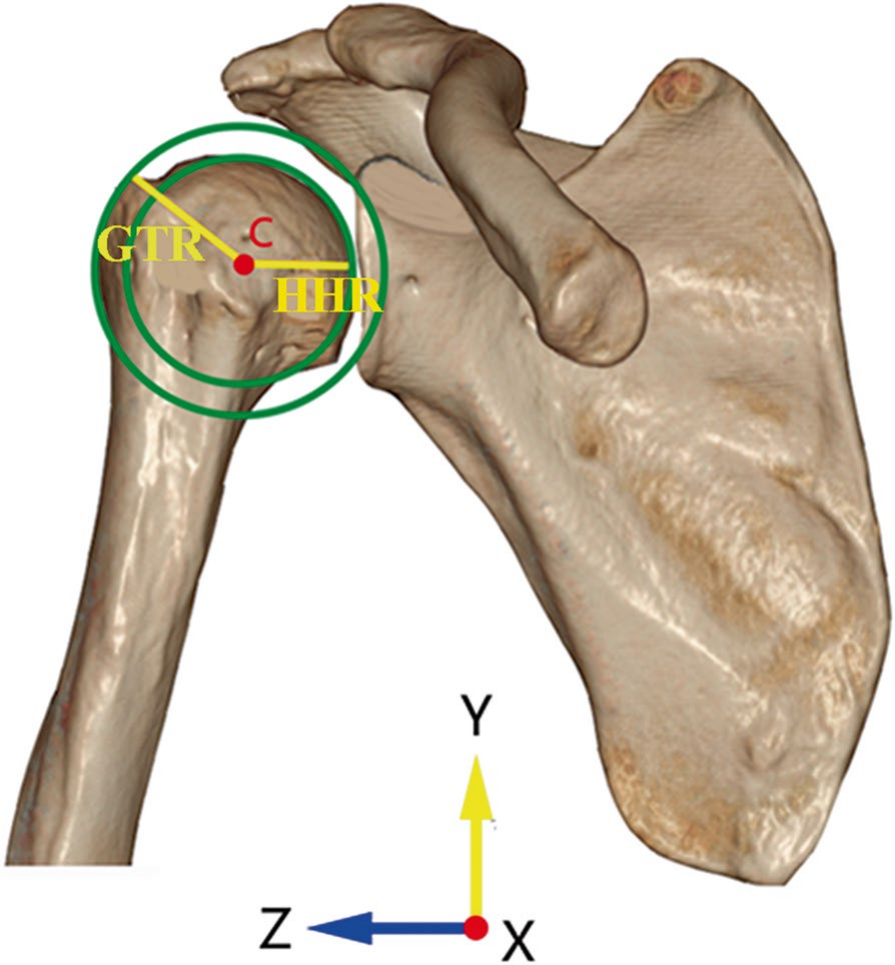
The double-circle system in anteroposterior view. The inside circle was the best-fit circle of the humeral head and the center was set as point C. The outside circle was a concentric circle with point C as the center and passed through the most lateral edge of the greater tuberosity. The radius of the inside circle was defined as the humeral head radius (HHR) and that of the outside circle was defined as the greater tuberosity radius (GTR). The ratio of HHR to GTR was defined as the double-circle radius ratio (DRR)

The DRR was a parameter to measure the superolateral extension of the greater tuberosity. In the true anteroposterior view, the best-fit circle of the humeral head was defined as the inside circle. The center of the inside circle was set as point C. Then we drew an outside concentric circle with the point C as the center and made this circle pass through the most superolateral edge of the greater tuberosity. The inside and outside circles formed the double-circle system (Fig. 2). Radiuses of the inside and the outside circles were defined as the humeral head radius (HHR) and the greater tuberosity radius (GTR), respectively. The ratio of GTR to HHR was defined as the DRR and a value over 1.38 was regarded as a risk factor of having a RCT [6].

#### (iii) Greater tuberosity angle (GTA)

**Fig. 3:**
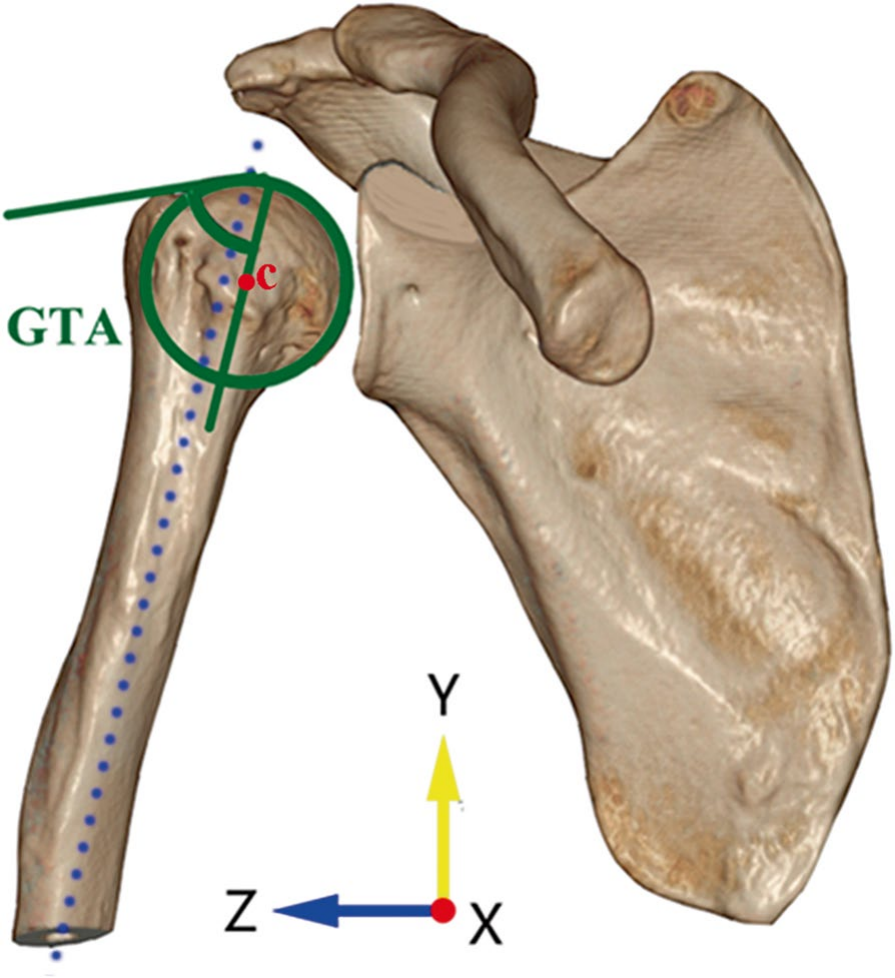
Measuring the greater tuberosity angle (GTA) in anteroposterior view. The center of the best-fit circle of the humeral head was set as point C. The angle by a line parallel to the humeral diaphyseal axis and passing the point C and another line connecting the upper border of the humeral head to the most superolateral edge of the greater tuberosity was measured as the GTA

The GTA was used to reflect the superolateral extension of the greater tuberosity in a perspective of angle. In the true anteroposterior view, the center of the best-fit circle of the humeral head was set as point C. The angle by a line parallel to the humeral diaphyseal axis and passing the point C and another line connecting the upper border of the humeral head to the most superolateral edge of the greater tuberosity was measured as the GTA (Fig. 3). A larger GTA was associated with a presence of RCTs and a GTA more than 70° was highly predictive in detecting RCTs [5].

#### (iv) Critical shoulder angle (CSA)

**Fig. 4:**
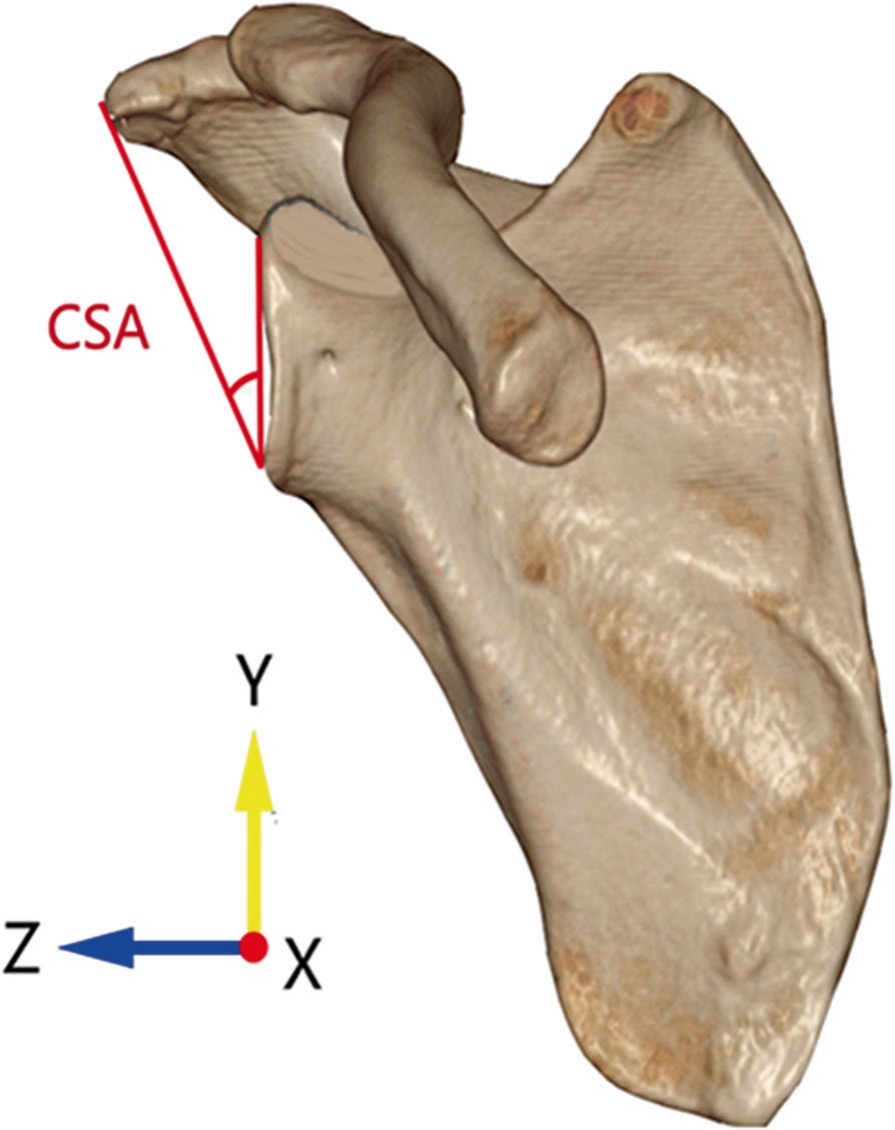
Measuring the critical shoulder angle (CSA) in anteroposterior view. The angle by a line connecting the inferior tip and the superior tip of the glenoid and another line connecting the inferior tip of the glenoid and the most lateral margin of the acromion was measured as the CSA

The CSA was used to measure the lateral extension of acromion. In the true anteroposterior view, the angle by a line connecting the inferior tip and the superior tip of the glenoid and another line connecting the inferior tip of the glenoid and the most lateral margin of the acromion was measured as the CSA (Fig. 4). The CSA was a widely accepted predictor for RCTs and a CSA more than 35° was significantly associated with the occurrence of RCTs [4].

#### (v) Acromion index (AI)

**Fig. 5:**
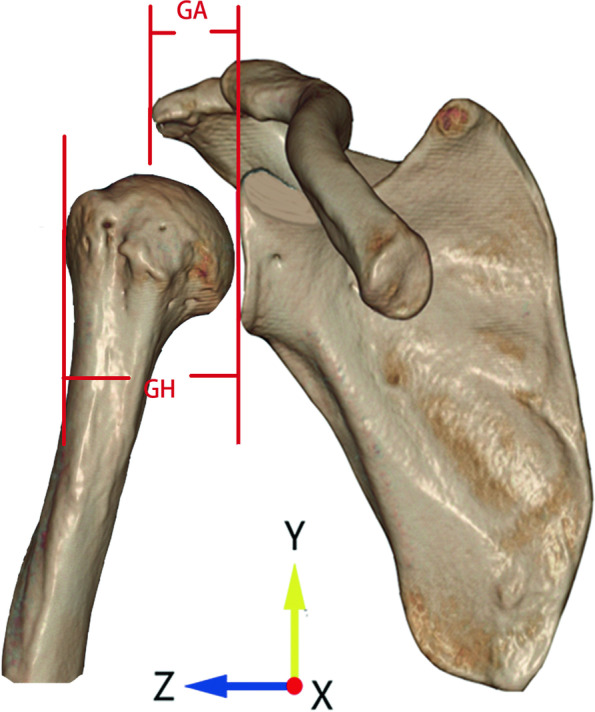
Measuring the acromion index (AI) in anteroposterior view. The distance from the glenoid plane to the lateral margin of acromion was measured as GA, and the distance from the glenoid plane to the lateral aspect of humeral head was measured as GH. The ratio of GA to GH was defined as AI

The AI was calculated to quantify the lateral extension of acromion. In the true anteroposterior view, the distance from the glenoid plane to the lateral margin of acromion was measured as GA, and the distance from the glenoid plane to the lateral aspect of humeral head was measured as GH (Fig. 5). The ratio of GA to GH was calculated as the AI. A larger value of AI was associated with a higher possibility of suffering from RCTs [3].

### Statistics

Statistical analysis was conducted with SPSS Statistics for Windows 24.0 software (IBM, Armonk, NY, USA). The normality of quantitative values was checked by Kolmogorov–Smirnov test. All quantitative values were reported as mean ± standard deviation. Quantitative variables were compared by independent samples t tests or Mann—Whitney U tests according to their normality of distribution, and qualitative variables were compared by chi-square tests to find significant differences between the RCT group and the control group. To increase accuracy of measurements, all variables were measured twice by the same author (QM) at two different time points and the average values were used for calculations. According to previous literatures and our pilot study, the standard deviations of the values of DRR, GTA, CSA and AI were presumed to be 0.08, 7°, 4° and 0.08, and the minimal clinical differences were considered as 0.05, 7°, 4° and 0.06 in the values of those predictors, respectively [3–6]. With the test of significant level as 0.05 and the power of test as 90%, a minimal sample size of 54 per group (2-tailed) was able to meet the statistical requirement. The intraclass correlation coefficient (ICC) of each measured value was presented with 95% confidence interval (CI). Binary logistic regression analysis was used to distinguish independent risk factors and calculate the increased odds of having a RCT, and construct discriminating combined models to further diagnose and predict RCTs. Receiver operating characteristic (ROC) curves were pictured to determine the overall diagnostic performance of the involved predictors and the combined models. For all tests a *p* value of < 0.05 was considered statistically significant.

## Results

### Enrollment of patients

**Fig. 6:**
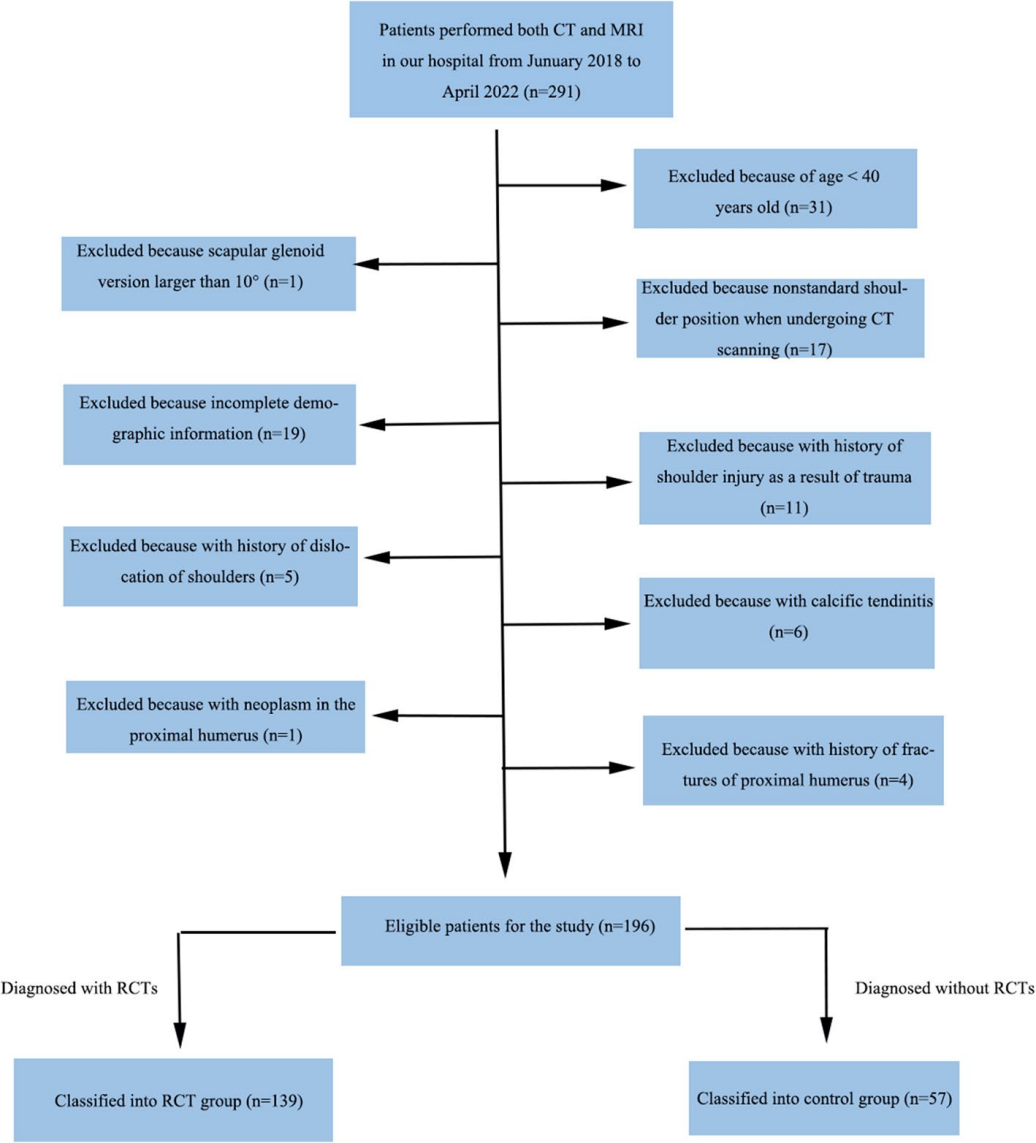
The flow chart of the screening for eligible patients

From January 2018 to April 2022 there were 291 patients performing both CT and MRI of shoulders in our hospital, and totally 95 patients were excluded according to the exclusion criteria, including 31 patients with age younger than 40 years old, one with a scapular glenoid version larger than 10°, seventeen with nonstandard shoulder position when undergoing CT scanning, nineteen with incomplete demographic information, eleven with history of shoulder injury as a result of trauma, five with history of dislocation of shoulders, six with calcific tendinitis, one with neoplasm in the proximal humerus, and four with history of fractures of proximal humerus. At last, 196 patients were enrolled in this study, of which 139 were classified into the RCT group and 57 into the control group (Fig. 6).

### Baseline information

The baseline information was presented in Table 1. The Kolmogorov–Smirnov tests indicated that the age (*p* = 0.034 and 0.200), stature (*p* = 0.042 and 0.046), body mass (*p* = 0.025 and 0.002) and BMI (*p* = 0.001 and 0.002) were not normally distributed in the RCT group and the control group. Hence, we used the Mann – Whitney U tests to find statistical differences between groups. The mean stature was 163.19 ± 8.47 cm in the RCT group and 167.23 ± 7.89 cm in the control group, and the difference was statistically significant (*P* = 0.002). The numbers of males and females in the RCT group were 50 and 89, and were 39 and 18 in the control group, respectively, showing a significant difference in gender proportion (*P* < 0.001). Other variables such as age, the ratio of affected side, body mass and BMI were found comparable between groups with all *p* values > 0.05.

**Table 1:** Baseline information

Demographic variable	RCT group (*n* = 139)	Control group (*n* = 57)	*P* value
Age, year	60.55 ± 9.63	59.39 ± 11.91	*0.327*
Gender, male to female, No	50/89	39/18	< *0.001*
Affected side, left to right, No	55/84	28/29	*0.219*
Stature, cm	163.19 ± 8.47	167.23 ± 7.89	*0.002*
Body mass, kg	68.03 ± 13.01	68.81 ± 13.64	*0.747*
BMI, kg/m^2^	25.49 ± 4.20	24.50 ± 3.76	*0.058*

*BMI* body mass index

### Comparison of predictors

The comparison of predictors was presented in Table 2. The Kolmogorov–Smirnov tests indicated that the CSA (*p* = 0.200 and 0.200), AI (*p* = 0.200 and 0.200), DRR (*p* = 0.200 and 0.200) and GTA (*p* = 0.200 and 0.200) were normally distributed in the RCT group and the control group, respectively. Hence, we used independent sample t tests to find statistical differences between groups. Significant differences were found in the values of CSA (35.36 ± 4.57 versus 31.41 ± 4.09°, *P* < 0.001), AI (0.69 ± 0.08 versus 0.63 ± 0.08, *P* < 0.001), DRR (1.43 ± 0.10 versus 1.31 ± 0.08, *P* < 0.001) and GTA (70.15 ± 7.38 versus 64.75 ± 7.91°, *P* < 0.001) between groups.

**Table 2:** comparison of predictors between groups

Predictors	RCT group (*n* = 139)	Control group (*n* = 57)	Mean difference (95% CI)	Effect size, Cohen’s d (95% CI)	*P* value
CSA, degree	35.36 ± 4.57	31.41 ± 4.09	3.95 (2.58 – 5.33)	0.89 (0.57 – 1.211)	< *0.001*
AI	0.69 ± 0.08	0.63 ± 0.08	0.05 (0.03 – 0.08)	0.75 (0.433 – 1.067)	< *0.001*
DRR	1.43 ± 0.10	1.31 ± 0.08	0.11 (0.09 – 0.14)	1.268 (0.935 – 1.601)	< *0.001*
GTA, degree	70.15 ± 7.38	64.75 ± 7.91	5.40 (3.06 – 7.74)	0.716 (0.4 – 1.033)	< *0.001*

*CSA* critical shoulder angle, *AI* acromion index, *DRR* double-circle radius ratio, *GTA* greater tuberosity angle, *CI* confidence interval

### Intraobserver reproducibility

The measured values of HHR, GTR, GTA, CSA, GA and GH at the first time were respectively 1.92 ± 0.20 cm, 2.74 ± 0.26 cm, 70.11 ± 7.42°, 35.54 ± 4.69°, 3.01 ± 0.40 cm and 4.43 ± 0.44 cm in the RCT group, and were 2.00 ± 0.16 cm, 2.61 ± 0.19 cm, 64.79 ± 7.97°, 31.58 ± 4.10°, 2.91 ± 0.39 cm and 4.61 ± 0.34 cm in the control group. The second measurements for these variables were respectively 1.92 ± 0.19 cm, 2.73 ± 0.26 cm, 70.19 ± 7.38°, 35.18 ± 4.47°, 3.03 ± 0.41 cm and 4.43 ± 0.44 cm in the RCT group, and were 1.99 ± 0.16 cm, 2.61 ± 0.20 cm, 64.71 ± 7.86°, 31.24 ± 4.10°, 2.92 ± 0.40 cm and 4.62 ± 0.33 cm in the control group. All measured variables were reliable and repeatable, with the ICC being 0.958 (95% CI, 0.945—0.968. *P* < 0.001) for HHR, 0.982 (95% CI, 0.976—0.986. *P* < 0.001) for GTR, 0.993 (95% CI, 0.991—0.995. *P* < 0.001) for GTA, 0.987 (95% CI, 0.973—0.992. *P* < 0.001) for CSA, 0.980 (95% CI, 0.973—0.985. *P* < 0.001) for GA and 0.987 (95% CI, 0.982—0.990. *P* < 0.001) for GH.

### Construction of combined models

Two predictors, one reflecting the lateral extension of acromion and another reflecting the superolateral extension of the greater tuberosity, were put together to perform the combined diagnosis of RCTs. With binary logistic regression analysis (having a RCT = 1, not having a RCT = 0), we got four combined models as following: Y_CSA+DRR_ = 0.228 × CSA + 14.529 × DRR – 26.619 (*P* < 0.001); Y_CSA+GTA_ = 0.211 × CSA + 0.1 × GTA – 12.846 (*P* < 0.001); Y_AI+DRR_ = 10.046 × AI + 15.822 × DRR – 27.385 (*P* < 0.001); Y_AI+GTA_ = 8.019 × AI + 0.106 × GTA – 11.527 (*P* < 0.001).

### Ability to diagnose rotator cuff tears

The performance of individual predictors and combined models to diagnose RCTs was determined by ROC curves. For individual predictors, the largest area under the curve (AUC) was observed in DRR as 0.823 (95% CI, 0.762 – 0.885, *P* < 0.001) with an applied cut off value of 1.37 (sensitivity, 71.9%; specificity, 82.5%). The CSA had an AUC of 0.746 (95% CI, 0.672 – 0.820, *P* < 0.001), and its cutoff value, sensitivity and specificity were 33.98°, 62.6% and 80.7%, respectively. The GTA had an AUC of 68.9 (95% CI, 0.604 – 0.773, *P* < 0.001) with a practical cutoff value of 67.58° (sensitivity, 61.2%; specificity, 70.2%). The smallest AUC was observed in AI as 0.668 (95% CI, 0.583 – 0.754, *P* < 0.001) with a best decisive cutoff value of 0.64 (sensitivity, 71.9%; specificity, 61.4%).

**Fig. 7:**
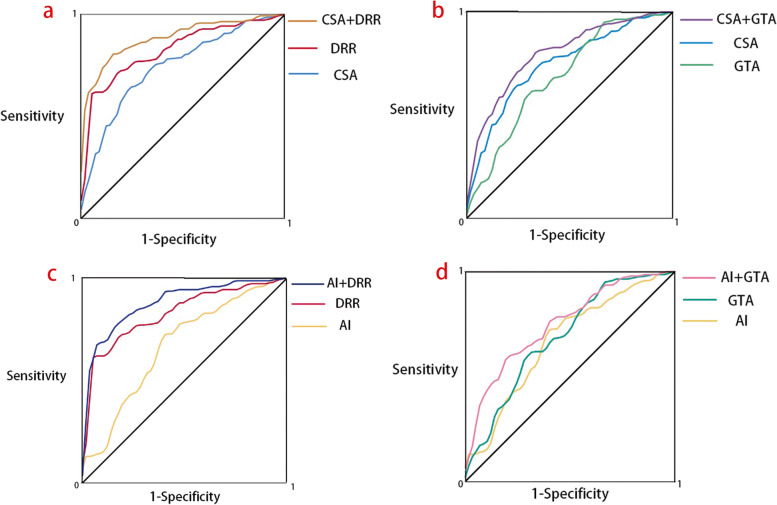
The receiver operating characteristic (ROC) curves present the diagnostic performance of the combined models and their contributors. **a** The combination of CSA and DRR. **b** The combination of CSA and GTA. **c** The combination of AI and DRR. (d) The combination of AI and GTA. No matter what combination it is, the combined models always show better diagnostic performance than their contributors

For combined diagnosis, the largest AUC was found in the model of CSA and DRR, which was 0.883 (95% CI, 0.836 – 0.930, *P* < 0.001) with a sensitivity of 77.7% and a specificity of 86.0% to diagnose RCTs. The model of AI and DRR had the second largest area (AUC = 0.872; 95% CI, 0.818 – 0.926, *P* < 0.001) with a sensitivity of 79.1% and a specificity of 82.5%, followed by the model of CSA and GTA (AUC = 0.797; 95% CI, 0.731 – 0.864, *P* < 0.001) with a sensitivity of 81.3% and a specificity of 66.7%, and the model of AI and GTA (AUC = 0.751; 95% CI, 0.678 – 0.824, *P* < 0.001) with a sensitivity of 59.0% and a specificity of 80.7%. No matter what combination it was, the combined model always had a larger AUC than either of its contributors (Fig. 7).

### Ability to predict rotator cuff tears

In this part, we defined the value of a predictor over its cutoff value as positive, and under its cutoff value as negative. According to the forementioned analysis of ROC curves, we considered the cutoff values of CSA, AI, GTA and DRR as 33.98°, 0.64, 67.58° and 1.37, respectively, to discriminate risk factors and predict the odds of developing a RCT in the present study. Via logistic regression analysis, we found a positive predictor was an independent risk factor of having a RCT, and a CSA > 33.98° led to an increased odds by 6.301 folds (95% CI, 2.495—15.914; *P* < 0.001), and an AI > 0.64 by 2.815 folds (95% CI, 1.182—6.703; *p* = 0.019), a GTA > 67.58° by 2.848 folds (95% CI, 1.202—6.751; *p* = 0.017) and a DRR > 1.37 by 11.191 folds (95% CI, 4.521—27.700; *P* < 0.001).

The predictive ability of the combined utilization of two predictors was also obtained by logistic regression analysis. Comparing to a patient with negative CSA and DRR, a patient with positive CSA and DRR had a 161.214—fold (95% CI, 20.354—1276.899; *P* < 0.001) increase in the risk of developing a RCT, while a positive CSA with a negative DRR, and a negative CSA with a positive DRR increased the odds by 6.871 folds (95% CI, 2.647—17.839; *P* < 0.001) and 11.159 folds (95% CI, 4.307 – 28.908; *P* < 0.001), respectively. As to the combination of CSA and GTA, the double positive, a positive CSA with a negative GTA, and a negative CSA with a positive GTA respectively increased the risk of having a RCT by 43.917 folds (95% CI, 9.533 – 202.317; *P* < 0.001), 6.889 folds (95% CI, 2.709—17.516; *P* < 0.001) and 3.904 folds (95% CI, 1.684 – 9.047; *p* = 0.001) comparing to double negative of those two predictors. With regard to the combination of AI and DRR, we found the odds of developing a RCT were increased by 63.467 folds (95% CI, 16.237 – 248.071; *p* = 0.001) with double positive of AI and DRR, and 4.421 folds (95% CI, 1.757 – 11.125; *p* = 0.002) with a positive AI and a negative DRR, and 12.400 folds (95% CI, 4.158 – 36.981; *P* < 0.001) with a negative AI and a positive DRR when comparing to the double negative. For the combination of AI and GTA, the double positive, a positive AI with a negative GTA, and a negative AI with a positive GTA resulted in increased odds by 20.169 folds (95% CI, 6.454 – 63.026; *P* < 0.001), 4.267 folds (95% CI, 1.762 – 10.333; *p* = 0.001) and 4.128 folds (95% CI, 1.582 – 10.772; *p* = 0.004), respectively, when comparing to the double negative. Details were presented in Table 3.

**Table 3:** Predictive ability of combined utilization of predictors

Combination	Positive or negative	Odds ratio	95% CI	*P value*
CSA and DRR	CSA( +) / DRR( +)	161.214	20.354—1276.899	< *0.001*
	CSA( +) / DRR(-)	6.871	2.647—17.839	< *0.001*
	CSA(-) / DRR( +)	11.159	4.307—28.908	< *0.001*
CSA and GTA	CSA( +) / GTA( +)	43.917	9.533—202.317	< *0.001*
	CSA( +) / GTA(-)	6.889	2.709—17.516	< *0.001*
	CSA(-) / GTA( +)	3.904	1.684—9.047	*0.001*
AI and DRR	AI( +) / DRR( +)	63.467	16.237—248.071	< *0.001*
	AI( +) / DRR(-)	4.421	1.757—11.125	*0.002*
	AI(-) / DRR( +)	12.400	4.158—36.981	< *0.001*
AI and GTA	AI( +) / GTA( +)	20.169	6.454—63.026	< *0.001*
	AI( +) / GTA(-)	4.267	1.762—10.333	*0.001*
	AI(-) / GTA( +)	4.128	1.582—10.772	*0.004*

( +), positive, means a value of a predictor > its cutoff value. (-), negative, means a value of a predictor < its cutoff value. *CSA* critical shoulder angle, *DRR* double-circle radius ratio, *AI* acromion index, *GTA* greater tuberosity angle, *CI* confidence interval

## Discussion

### Comparison of predictors between groups

The mean values of CSA, AI, DRR and GTA in the RCT group were all significantly larger than those in the control group, showing distinguishable morphological differences of both acromion and the greater tuberosity between patients with or without RCTs. The CSA was first proposed by Moor et al. in 2013 and had been widely accepted as a valuable indicator for the progression of RCTs with an average value ranging from 35° to 39° in shoulders with RCTs and from 32° to 37° in shoulders without teared tendons [4, 10–13]. In our study, the mean values of CSA were 35.36° in the RCT group and 31.41° in the control group, which was compatible with those revealed in previous researches. The AI was another predictor involved in our study, which was proposed even earlier than CSA and was found a value of > 0.68—0.73 was significantly associated with a presence of RCTs [3, 14–16]. In agreement with the previous studies, the average values of AI in our study were 0.69 in the RCT group and 0.63 in the control group. Theoretically, the larger the lateral extension of acromion is, the higher ascending force the deltoid generates, pulling humeral head upward to impinge against acromion [3]. Another hypothesis also indicated that the injured cuff due to intrinsic factors might not be able to oppose the superior pull of the deltoid, resulting in a cranial decentralization of humeral head and a secondary subacromial impingement to accelerate the breakdown of the affected tendon [17].

Unlike CSA and AI, the impact of the superolateral extension of the greater tuberosity on the development of RCT was just recognized in recent years. A classical predictor, the GTA, was created in 2018 and a mean value of > 70—72° significantly associated with developing a RCT [5, 18]. In our study, the mean values of GTA were 70.15° in the RCT group and 64.75° for the control, comparable to the previous findings. DRR is another practical parameter to diagnose and predict RCT with a value over 1.38 considered as a risk factor of having a RCT [6], which was in accordance with our results in the present study. The superolateral extension of the greater tuberosity narrows the subacromial space and makes the force vector of supraspinatus more divergent from deltoid, increasing the load on supraspinatus when lifting the upper limbs [5]. Although the whole pathological process of RCTs is not completely clear, we believe it occurs due to the contribution from the acromion and the greater tuberosity together.

### Diagnostic ability

The diagnostic ability was explored via ROC curves. For individual predictor, DRR had the largest AUC (0.823), followed by CSA (0.746), GTA (0.689) and AI (0.668), indicating the DRR had the strongest power to diagnose RCTs. To rank the sensitivity of diagnosis from highest to lowest, DRR and AI with the same sensitivity of 71.9% got the top place, followed by CSA with a sensitivity of 62.6%, and GTA with a sensitivity of 61.2%. In terms of the specificity of diagnosis, DRR had the highest specificity as 82.5%, followed by CSA being 80.7%, GTA being 70.2% and AI being 61.4% in order from highest to lowest. Based on a comprehensive consideration of diagnostic performance, we recommend the DRR, which has the highest sensitivity and specificity among those predictors, as the best choice to diagnose RCTs. To reduce the false positive rate, AI could be an alternative because of its high sensitivity in diagnosing RCTs. To minimize the rate of false negative, CSA could be an alternative because it has a comparable specificity to that of DRR.

Among the combined models, CSA and DRR together had the largest AUC being 0.883, followed by the combination of AI and DRR being 0.872, CSA and GTA being 0.797 and AI combined with GTA being 0.751, showing the combination of CSA and DRR was the most superior approach to diagnose RCTs. From a perspective of the sensitivity for diagnosis, CSA together with GTA had the highest sensitivity of 81.3% and AI together with DRR had the second highest sensitivity of 79.1%, followed by the combination of CSA and DRR being 77.7% and AI together with GTA being 59.0%. From another perspective of the specificity for diagnosis, CSA combined with DRR had the highest specificity of 86.0%, and the combination of AI and DRR contributed to a second highest specificity of 82.5%, followed by AI together with GTA being 80.7% and CSA combined with GTA being 66.7%. According to the diagnostic performance of those combined models, we recommend CSA together with DRR as the best combination to diagnose RCTs. To reduce the rate of false positive, the combination of CSA and GTA is a better choice because it has the highest diagnostic sensitivity. To reduce the rate of false negative, AI combined with DRR could be an alternative because of its comparable specificity to that of the combination of CSA and DRR.

Both as parameters to assess the lateral extension of acromion, the diagnostic ability of CSA and AI had been discussed in previous work. In Moor et al.’s analysis with a cohort of 51 patients in the RCT group and 51 in the control group, CSA was reported to have a sensitivity of 80% and a specificity of 75% for differentiating RCTs and normal patients, while AI was reported to have a slightly lower sensitivity of 78% and a specificity of 71% [10]. In another research where the diagnostic performance of CSA and AI was compared, the authors found CSA had a larger AUC being 0.86 than that of AI being 0.80, concluding CSA was more superior than AI for diagnosing RCTs [19]. In the present study, the AUC of CSA was larger than that of AI (0.746 versus 0.668), proving a more outstanding diagnostic ability in CSA and showing satisfied consistence with previous results. Unfortunately, literatures to discuss the diagnostic performance of GTA and DRR were rare because they were just created lately. As the requirement for the minimal sample size had been met and ICCs showed good agreement for all measurements, we believed our results were convincible and reliable with a good repeatability.

### Predictive ability

The predictive ability was assessed by logistic regression analysis. With the values of the involved predictors over their respective cutoff values, CSA increased the risk of having a RCT by 6.301 folds, and AI by 2.815 folds, GTA by 2.848 folds and DRR by 11.191 folds. Therefore, we concluded the DRR had the strongest predictive ability to detect RCTs, follow by the CSA as an alternative.

In another aspect, among the combined models, when both the values of the predictors were larger than their respective cutoff values, CSA together with DRR increased the odds of developing a RCT by 161.214 folds, followed by AI together with DRR by 63.467 folds, CSA together with GTA by 43.917 folds, and AI together with GTA by 20.169 folds comparing to both the values lower than their cutoff values. As a result, we recommended the combined utilization of CSA and DRR, which had a much better predictive ability than the rest, as a first-line screening method for early prediction of RCTs.

Previous studies usually used a single predictor to predict RCTs, and the CSA, DRR, GTA and AI were regarded as independent risk factors for a presence of a RCT with increased odds of 10.8 folds [10], 11.252 folds [6], 93 folds [5], and 1.998 folds [20], respectively. However, controversy remains in the pragmatic clinical application of those parameters. In Pandey et al.’s research, they found a significant difference in the value of AI between the full-thickness RCT group and the control group, but the stepwise logistic regression rejected the AI as a predictor for the occurrence of a RCT [13]. A similar dilemma was observed in the GTA, as it significantly differed between groups but failed to be eligible for a predictor of full-thickness degenerative supraspinatus tear [18]. In our study, the AI and the GTA were both qualified as independent risk factors, but their predictive abilities were a little weaker, only increasing the odd of having a RCT by approximately three folds. An appropriate improvement to increase their detectability was necessary in the situation.

### Clinical recommendation

With the recent economic downturn due to the pandemic of the COVID-19, how to reduce unnecessary medical expenditure is a substantial question for potential patients complaining with shoulder discomfort. Although MRI is the most common screening tool to detect RCTs with an extremely high diagnostic accuracy of 89.09% [21], it costs a lot and may become a source of economic burden in most situations. An alternative solution is to perform the diagnosis with the forementioned predictors in X-ray imaging, which is cost-efficient and much cheaper than MRI. In Fig. 6, we can tell that no matter what combination it is, the combined model always shows a better diagnostic performance than either of its contributors, improving the AUC from 0.668—0.823 to 0.751—0.883, and extending the upper boundary of diagnostic sensitivity from 71.9% to 81.3%, and the diagnostic specificity from 82.5% to 86.0%. As a consequence, we suggest to take the morphological characteristics of both acromion and the greater tuberosity into account and use a combined model, especially the combination of CSA and DRR, to diagnose a RCT.

Another focus issue is the early detection and prevention of RCTs in patients with symptomatic shoulders but a negative MRI. A single predictor only increases the odds of developing a RCT by 2.815—11.191 folds, however, the risk increases sharply with a reconsideration from a view point of combined models. In Table 3 where the value of a predictor over its cutoff value is defined as positive and under is defined as negative, we can see that among all combinations, double positive predictors result in a dramatical and enormous increasement (20.169—161.214 folds) in the risk of having a RCT, which is far beyond that by a positive one together with a negative one (3.904—12.400 folds). Hence, we recommend the combined utilization, especially the combination of CSA and DRR, as a better approach to predict the occurrence of a RCT rather than a single predictor. For a patient with a symptomatic shoulder but MRI revealing no injury in rotator cuffs, we should remind him of high possibility of suffering from a RCT in the future if the combined prediction indicates an increased risk of having a RCT.

### Strength of the study

The application of predictors to diagnose and predict RCTs had been explored for a long period of time but almost all researches were performed with a single predictor, and discussions about the overall diagnostic and predictive performance of combined models were very rare. To our best acknowledge, the present study was the first to have a detailed discussion on the diagnostic and predictive ability of combined models, providing a substantial supplement in the relative field. Another highlight of the study was that we picked four predictors to participate in our research, two to assess the lateral extension of the acromion and another two to evaluate the superolateral extension of the greater tuberosity, producing totally four combinations to verify our hypothesis. At last, all combinations showed a more superior diagnostic and predictive performance than using a single predictor, making our conclusion more convincible.

### Limitations

There are some limitations in this study. First, although the requirement for the minimal sample size in each group has been met, the sample size is still not large enough, especially that of the control group. The imbalance of numbers of patients in two groups may contribute to a bias in the results. A larger cohort is needed to further verify our findings. The second limitation is that all the measurements were performed on 3D models established via CT scanning, leaving disadvantages of high costs and complicated manipulating procedures compared to X-ray images. Unfortunately, standard true anteroposterior view of shoulder on X-ray imaging is lacked in our hospital. Therefore, we chose to continue our research by CT scanning. Further studies to discuss the performance of the combined model on X-ray radiology are essential to promote it to be a widespread and cost-efficient application in clinical practice. The third limitation is that all the values associated with the greater tuberosity were measured in coronal plane and did not take into account the anteroposterior relationship between the greater tuberosity and the humeral head, which may potentially cause bias to the practicability of our findings. Finally, only 67% of the eligible patients (196 out of 291) were enrolled in the study because of the strict exclusion criteria, leaving a disadvantage of reduced external validity. Maybe well-designed prospective research could be a solution for this weakness.

## Conclusion

The combined utilization of predictors is a better approach to diagnose and predict RCTs than using a single predictor. With a comprehensive consideration on the diagnostic and predictive performance of the combined models, we conclude that the CSA together with the DRR present the strongest detectability for a presence of RCTs.

## Data Availability

Datasets are available through the corresponding author upon reasonable request.

## Abbreviations

RCT: Rotator cuff tear
AI: Acromion index
CSA: Critical shoulder angle
GTA: Greater tuberosity angle
DRR: Double-circle radius ratio
MRI: Magnetic resonance imaging
CT: Computed tomography
3D: Three-dimensional
HHR: Humeral head radius
GTR: Greater tuberosity radius
ICC: Intraclass correlation coefficient
CI: Confidence interval
ROC: Receiver operating characteristic
AUC: Area under curve
